# Analyses of Electric Field-Induced Phase Transformation by Luminescence Study in Eu^3+^-doped (Na, K)_0.5_Bi_0.5_TiO_3_ Ceramics

**DOI:** 10.3390/ma13061347

**Published:** 2020-03-16

**Authors:** Liang Zeng, Ji Zhou

**Affiliations:** State Key Laboratory of New Ceramics and Fine Processing, School of Materials Science and Engineering, Tsinghua University, Beijing 100084, China; zeng-l15@mails.tsinghua.edu.cn

**Keywords:** luminescence lifetime, Judd–Ofelt theory, phase transformation, quantitative analyses, ferroelectric ceramics, decay kinetics

## Abstract

Most analyses of phase transformations detected by rare earth ions are based on the luminescence spectrum, while in this study we focus on the luminescence decay processes. We prepared Eu^3+^-doped (Na, K)_0.5_Bi_0.5_TiO_3_ ceramics and studied their phase structure before and after poling by luminescence spectra, decay curves, and X-ray diffraction (XRD). Luminescence spectra indicated that electric fields induced a transformation in (Na_0.8_, K_0.2_)_0.5_Bi_0.497_Eu_0.003_TiO_3_ (NKBET20) ceramic from tetragonal to rhombohedral phase (R phase). Based on the decay kinetics and the Judd–Ofelt theory, decay curves were shown to identify the fraction of the transformation quantitatively. The data from decay curves suggest that with electric fields increasing from 0 to 50 kV/cm, the R phase fraction increases from about 23 to 89% and the tetragonal phase (T phase) fraction decreases from about 77 to 11%. XRD Rietveld analyses further confirmed the results. In this work, the analyses of the phase fractions are simplified by the monoexponential decay of the pure phases and the biexponential decay of the mixed phase, showing an easy and inexpensive way of studying the phase structures of the materials.

## 1. Introduction

Investigations on rare-earth (RE) ions-doped ferroelectric ceramics have attracted significant interest because of the interactions of electrical and luminescence properties [1,2,3]. On one hand, RE ions doped in ferroelectric materials show efficient emissions, while the excellent electrical properties of the ferroelectric materials could be retained or some improved [4,5]. This makes the ferroelectric materials doped with RE ions multifunctional materials [6,7]. For instance, it is reported that electronic-mechanic-optical coupling can be achieved in the BaTiO_3_-CaTiO_3_: Pr materials [8]. On the other hand, when RE ions are doped in dilute concentration, they can be utilized as spectroscopic probes to detect phase structures of the hosts [9,10]. The phase symmetry of the hosts has important influences on the luminescence properties of doped RE ions, such as crystal-field splitting, luminescence lifetime, and emission efficiency. Thus, the luminescence data could reflect the information of the phase symmetry of the hosts [11]. Among all the RE ions, Eu^3+^ ions have gained significant concerns for their simple electronic configuration. The levels D05 and F07 of Eu^3+^ ions are the main emissive level and the ground level, respectively, which are nondegeneration, simplifying the analyses greatly [12]. Besides, the transition D05→F27 originating from an electric diploe (ED) is hypersensitive to its environments, while the transition D05→F17 originating from a magnetic dipole (MD) is independent of its environments [13,14]. These different responses of the two transitions to the environments make Eu^3+^ ions a very useful probe. Eu^3+^ ions have been utilized to detect phase structures [15], phase transformations [9], etc. However, most analyses of the reported results were based on the luminescence spectra, the luminescence decay curves of Eu^3+^ ions are seldom concerned. According to Judd–Ofelt theory [11], the probabilities for radiative transitions and nonradiation processes determine the lifetime of an emissive level, which can be given by

(1) $$\frac{1}{\tau }=\sum_{J^{\prime }}A(\Psi J,\Psi J^{\prime })+W$$

Here, τ is the lifetime of level J; $A(\Psi J,\Psi J^{\prime })$ is the radiative probability of the transition $\Psi J\to \Psi J^{\prime }$; the summation is for transitions which terminate on final level $J^{\prime }$; and W is the nonradiative probability. As we can see, the radiative probabilities that determine the luminescence spectra would influence the luminescence lifetime greatly. Thus, as an intrinsic property, the luminescence lifetime could also give the information of the hosts. In addition, unlike the absolute luminescence intensity, which is affected by the conditions of the measurements and samples, fitted from the decay curves, the luminescence lifetime of the emissive level is a stable parameter for measurements. Here, we focus on the analyses of the luminescence decay curves of Eu^3+^ ions and use them as tools to probe the phase structure.

As functional materials, ferroelectric ceramics are widely applied in electromechanical devices. In general, the optimal electrical performance of ferroelectric ceramics could be realized with multi-phases coexistence like morphotropic phase boundary (MPB) [16,17]. Under such conditions, the Gibbs energy gap of phases is small [18], indicating that outer conditions, such as pressures and electric fields, can easily cause phase transformations [19,20,21]. In particular, electric field-induced phase transformations are of great concern because they can control the performance of ferroelectric ceramics via voltages [22,23].

In this work, we fabricated (Na_0.8_, K_0.2_)_0.5_Bi_0.497_Eu_0.003_TiO_3_ (NKBET20) ceramics and investigated the phase structures of NKBET20 ceramics by luminescence spectra, decay curves, and XRD, before and after poling. (Na_1−x_, K_x_)_0.5_Bi_0.5_TiO_3_ (NKBT100x) ceramics have been widely studied due to their excellent electrical properties, which crystallizes R phase in the composition rich in Na_0.5_Bi_0.5_TiO_3_ and T phase in the composition rich in K_0.5_Bi_0.5_TiO_3_ [24,25]. When x is in the range of 0.16–0.2, NKBT100x ceramics form MPB, with R and T phases coexisting [26]. This work reveals that electric fields induced a transformation in NKBET20 ceramics from T to R phase. Besides, luminescence decay curves were shown to detect the phase transformation quantitatively, according to the decay kinetics and the Judd–Ofelt theory. Additionally, investigations by X-ray Rietveld analyses correlate well with the presented data from the decay curves analyses.

## 2. Materials and Methods

(Na_1−x_, K_x_)_0.5_Bi_0.497_Eu_0.003_TiO_3_ (x = 0.1, 0.2 and 0.3, abbreviated as NKBET10, NKBET20 and NKBET30, respectively) ceramic pellets were fabricated by solid reaction method. Detailed preparations were shown elsewhere [15]. By ion sputtering (SBC-12, KYKY Technology Co., Beijing, China), golden electrodes were used upon both sides of the pellets. Next, the pellets were poled in silicone oil under various dc electric fields for 0.5 h. After golden electrodes corroded by aqua regia, XRD and luminescence measurements were executed. The X-ray diffractometer (D/max-2500H, Rigaku, Tokyo, Japan) was used for XRD measurements. The working voltage and current of the diffractometer were set at 40 kV and 150 mA, respectively. The patterns were scanned from 20° to 120° with interval of 0.01°. The luminescence properties were recorded by a spectrophotometer (FLSP920, Edinburgh Instruments, Livingston, UK). The excitation wavelength was set at 525 nm. For luminescence spectra, the monitored luminescence ranged from 570 to 720 nm, and for decay curves, the monitored luminescence was set at 592 nm.

## 3. Results and Discussions

**Figure 1:**
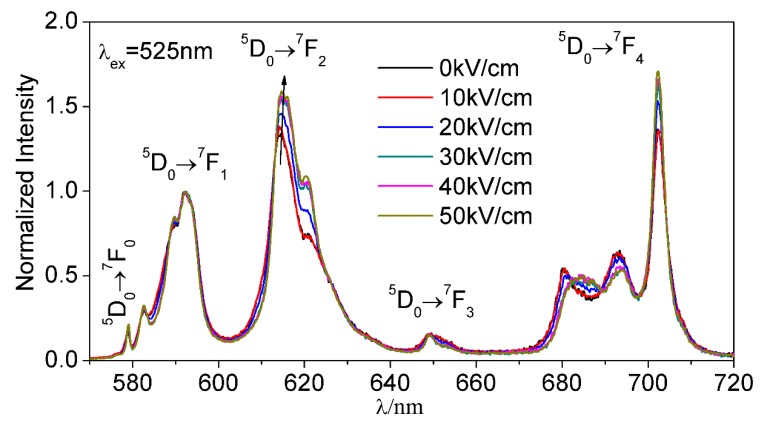
The luminescence spectra of (Na_0.8_, K_0.2_)_0.5_Bi_0.497_Eu_0.003_TiO_3_ (NKBET20) ceramics poled under various electric fields. The excitation wavelength was set at 525 nm; the black arrow indicates the variation of the D05→F27 transition.

The luminescence spectra of NKBET20 ceramics poled under various electric fields are shown in Figure 1. The luminescence spectra in wavelength ranges of 570–720 nm consist of five main different emission bands, which originate from transitions D05→F07, D05→F17, D05→F27, D05→F37, and D05→F47 of Eu^3+^ ions, respectively, [11]. Since the transition D05→F17 is insensitive to its environments, the peak intensity of this transition is normalized for better comparison, while the transition D05→F27 is sensitive to its environments [11]. Thus, the intensities of MD and hypersensitive transitions indicate the environments of Eu^3+^ ions. Unpoled NKBET20 ceramics present a phase structure with R phase and T phase coexisting, while electric fields could induce a phase transformation, which are also reflected on the luminescence spectra [27]. As shown in Figure 1, with electric fields increasing, the intensities of the transition D05→F27 increase, indicating a phase transformation from T to R phase, since a lower symmetry contributes to a stronger intensity for a “hypersensitive transition” [11,15].

**Figure 2:**
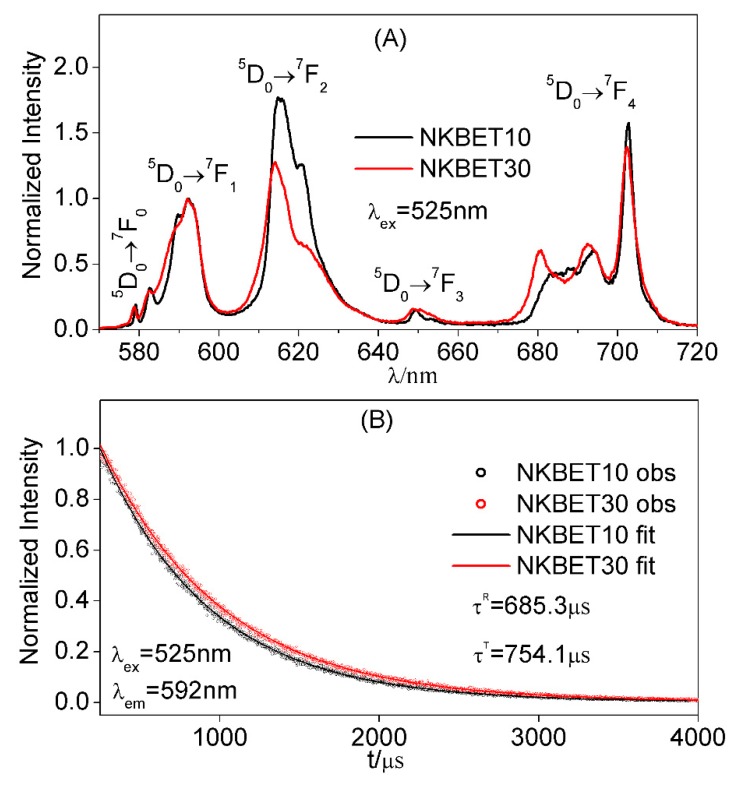
(**A**) The luminescence spectra of (Na_0.9_,K_0.1_)_0.5_Bi_0.497_Eu_0.003_TiO_3_ (NKBET10) and (Na_0.7_,K_0.3_)_0.5_Bi_0.497_Eu_0.003_TiO_3_ (NKBET30) ceramics. (**B**) The luminescence decay curves of NKBET10 and NKBET30 ceramics, fitted with a monoexponential function I(t)=I(0)exp(−t/τ).

For (Na_1−x_, K_x_)_0.5_Bi_0.497_Eu_0.003_TiO_3_ (NKBET100x) ceramics, Eu^3+^ ions present different emission properties when distributed in different phases [15]. Figure 2A shows the luminescence spectra of NKBET10 and NKBET30 compositions as references of R phase and T phase. As we can see, the shapes of the luminescence spectra of NKBET10 and NKBET30 ceramics differ greatly. These differences indicate that the radiative properties of the transitions D05→FJ7 of Eu^3+^ ions in the two phases are different, which would further govern the decay time (seen in the Supplementary Materials). The luminescence decay curves of NKBET10 and NKBET30 ceramics are shown in Figure 2B, fitted with a monoexponential function. The luminescence lifetimes of Eu^3+^ ions in NKBET10 and NKBET30 ceramics are found be to 685.3 μs and 754.1 μs, respectively. In our earlier work [27], we show that using the luminescence spectra of NKBET10 and NKBET30 ceramics as references, the variations of luminescence spectra could be further utilized to quantitatively calculate the phase transformations in NKBET20 ceramics. Similar to the luminescence spectra, information about phase transformations could be extracted from luminescence decay curves.

As Eu^3+^ ions present different emission properties when distributed in different phases, the emission properties of NKBET20 ceramics with R and T phases coexistence comprise the contributions of Eu^3+^ ions in the two phases. This means that in the stable states,

(2) $$I=I^{R}+I^{T}$$

where I is the total luminescence intensity of NKBET20 ceramics, and superscripts R and T represent the contributions from Eu^3+^ ions in R and T phases, respectively.

The luminescence intensity of the radiative transition is proportional to the population of the according emissive level [28,29]; then for the transition D05→F17 we obtain

(3) $$I_{0\to 1}=A_{0\to 1}^{R}N^{R}+A_{0\to 1}^{T}N^{T}$$

where $I_{0\to 1}$ is the total luminescence intensity of the transition D05→F17 in counts per second (s^−1^)) of NKBET20 ceramics; $A_{0\to 1}$ and N are the radiative probability of the transition D05→F17, and the population of the level D05, respectively.

While in the decay processes, the two different kinds of Eu^3+^ ions in NKBET20 ceramics decay differently, which means the decay follows a biexponential function, then

(4) $$I_{0\to 1}(t)=A_{0\to 1}^{R}N^{R}\exp(-t/\tau^{R})+A_{0\to 1}^{T}N^{T}\exp(-t/\tau^{T})$$

where $I_{0\to 1}(t)$ is the total emission intensity of the transition D05→F17 of NKBET20 ceramics at time t. Here, we used NKBET10 and NKBET30 compositions as references of R and T phases respectively, and $\tau^{R}$ and $\tau^{T}$ are 685.3 μs and 754.1 μs, respectively.

From the Judd–Ofelt theory [11], $A_{0\to 1}$ can be calculated by

(5) $$A_{0\to 1}=\frac{64\pi^{4}v_{1}^{3}}{3h}D_{1}n^{3}$$

where h is the Planck constant, and $v_{1}$ and $D_{1}$ are the average wavenumber and the dipole strength of the transition, respectively. Since the MD transition D05→F17 is insensitive to its environments, $v_{1}$ and $D_{1}$ nearly remain unchanged for Eu^3+^ ions in different phases. n is the refractive index of the hosts, neglecting the difference of n between phases in NKBET20 ceramics (seen in Figure S1), then

(6) $$A_{0\to 1}^{R}=A_{0\to 1}^{T}$$

The population of D05 level can be described as [15]

(7) $$N=\frac{8\pi^{3}}{3h^{2}c}n\tau \rho SXC$$

where c is the speed of light, ρ is the energy density at the wavenumber of the transition D15←F07, S is the dipole strength of the transition D15←F07, X is the fractional thermal population of the level F07, and C is the total number of Eu^3+^ ions. ρ is related to the power of the excitation light; S is the dipole strength of the MD transition D15←F07; X is estimated be to 65% at room temperature [11]; thus, ρ, S, and X nearly remain the same for Eu^3+^ ions in different phases.

If we define $\alpha^{R}$ and $\alpha^{T}$ as the volume fractions of R and T phases in NKBET20 ceramics, respectively, $\alpha^{R}C$ and $\alpha^{T}C$ will be the number of Eu^3+^ ions in R and T phases, respectively. Neglecting the difference of n between phases, from above analyses we obtain

(8) $$N^{R}=\frac{8\pi^{3}}{3h^{2}c}n\tau^{R}\rho SX\alpha^{R}C$$

(9) $$N^{T}=\frac{8\pi^{3}}{3h^{2}c}n\tau^{T}\rho SX\alpha^{T}C$$

Equations (8) and (9) describe the relationship of the phase fraction and the population of the D05 level, which would further influence the decay processes. In order to figure out the phase fractions, we define

(10) $$B=\frac{A_{0\to 1}^{R}N^{R}}{A_{0\to 1}^{T}N^{T}}$$

Combined with Equations (6), (8), and (9), Equation (10) can be rearranged as

(11) $$B=\frac{\tau^{R}\alpha^{R}}{\tau^{T}\alpha^{T}}$$

From Equation (11) together with

(12) $$\alpha^{R}+\alpha^{T}=1$$

we can figure out the volume fractions of R and T phases,

(13) $$\alpha^{R}=\frac{B\tau^{T}}{\tau^{R}+B\tau^{T}}$$

(14) $$\alpha^{T}=\frac{\tau^{R}}{\tau^{R}+B\tau^{T}}$$

where B can be fitted from the decay process, and $\tau^{R}$ and $\tau^{T}$ are 685.3 μs and 754.1 μs, respectively, using NKBET10 and NKBET30 ceramics as references, respectively.

**Figure 3:**
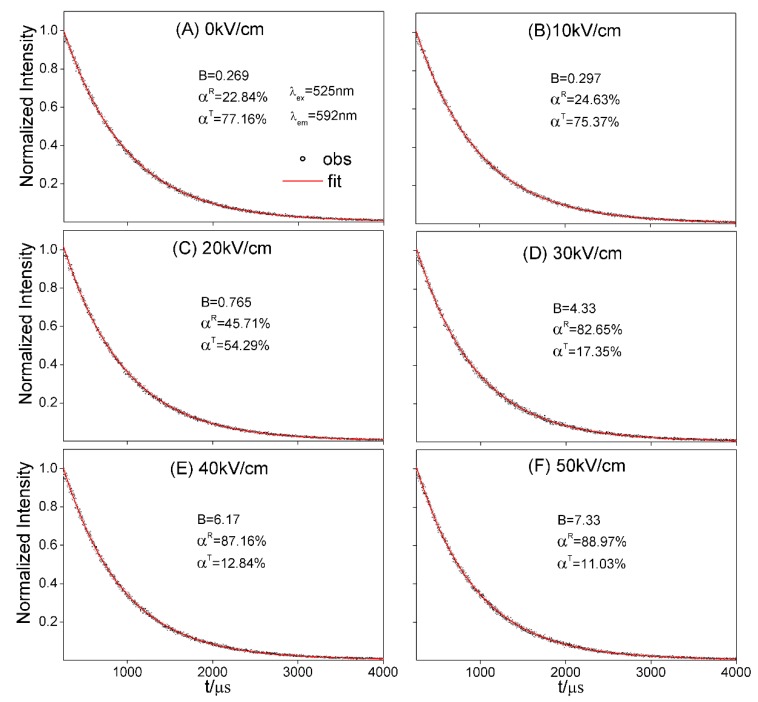
The decay curves of NKBET20 ceramics poled under various electric fields, fitted with a biexponential function, $I_{0\to 1}(t)=A_{0\to 1}^{R}N^{R}\exp(-t/\tau^{R})+A_{0\to 1}^{T}N^{T}\exp(-t/\tau^{T})$, where $\tau^{R}$ and $\tau^{T}$ are 685.3 μs and 754.1 μs respectively, using NKBET10 and NKBET30 ceramics as references. (**A**) 0 kV/cm, (**B**) 10 kV/cm, (**C**) 20 kV/cm, (**D**) 30 kV/cm, (**E**) 40 kV/cm, (**F**) 50 kV/cm.

Above analyses reveal that the phase fractions of NKBET20 ceramics could be extracted from the decay processes, and here we used the method to identify the fraction of the phase transformation in NKBET20 ceramics induced by electric fields. The decay curves of NKBET20 ceramics poled under various electric fields are shown in Figure 3, fitted with a biexponential function.

After B were fitted from the decay processes, solving Equations (13) and (14), we could figure out the fractions of the phase transformation.

**Figure 4:**
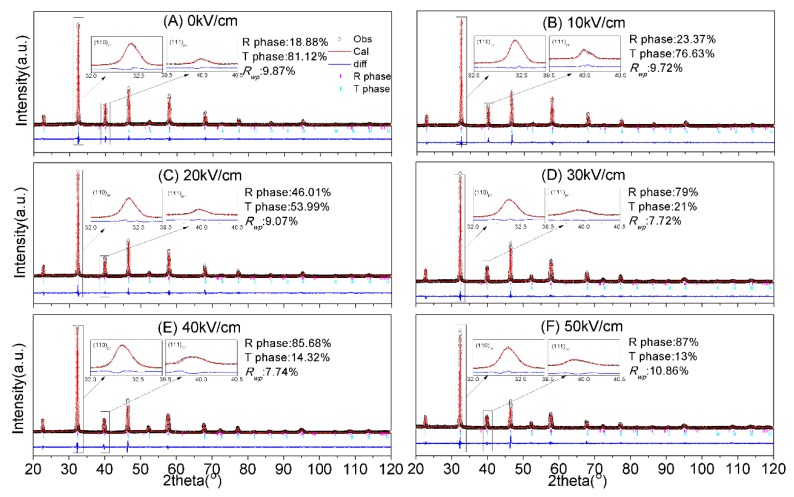
XRD Rietveld analyses of NKBET20 ceramics poled under various electric fields. (**A**) 0 kV/cm, (**B**) 10 kV/cm, (**C**) 20 kV/cm, (**D**) 30 kV/cm, (**E**) 40 kV/cm, (**F**) 50 kV/cm. Adapted from our earlier work [27].

Additionally, XRD Rietveld analyses were carried out by GSAS [30], in which space groups R3c and P4mm were used [31,32]. Figure 4 depicts the refinement patterns, and Table 1 summarizes the refinement parameters, from which it can be concluded that the refinements were well fitted. The crystal structure parameters are shown in Tables S1 and S2.

**Figure 5:**
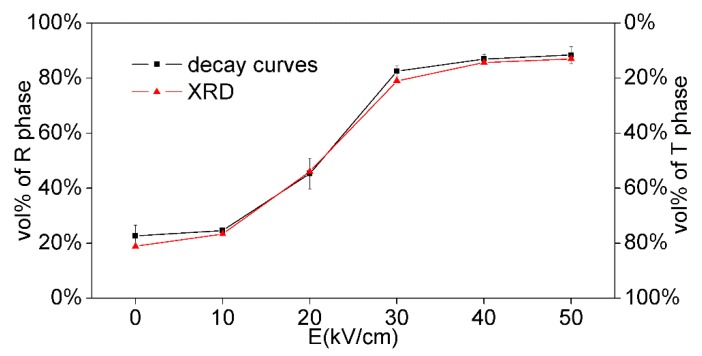
Variations of the phase fractions of NKBET20 ceramics poled under various electric fields from decay curves and XRD analyses.

The phase fractions of NKBET20 ceramics poled under various electric fields from decay curves and XRD Rietveld analyses are shown in Figure 5. From the data given by decay curves, the R phase fractions increase from about 23% to 89% and the T phase fractions decrease from about 77% to 11% with the electric fields increasing from 0 to 50 kV/cm, which agrees with the XRD Rietveld analyses. Compared to the XRD Rietveld analyses, which demands high-quality X-ray diffractometer and precise data, the decay curves method shows a fast and simple procedure. Besides, the experiment set ups for decay curves is easy to build, which can be home-made for various demands, like electric fields module, pressures module, and temperature module. We present that the decay curve method could be used to analyze the phase transformation induced by electric fields in this work, yet it can be used in other occasions of phase transformations.

## 4. Conclusions

In summary, the luminescence spectra indicated a transformation from T to R phase in NKBET20 ferroelectric ceramics after poling. Further, based on the decay kinetics together with the Judd–Ofelt theory, the decay curves of the NKBET20 ceramics were shown to identify the fraction of the phase transformation, using the luminescence lifetime of NKBET10 and NKBET30 ceramics as references. The data from the decay curves show that the R phase fraction increases from about 23% to 89% and the T phase fraction decreases from about 77% to 11%, with the electric fields increasing from 0 to 50 kV/cm. Additionally XRD Rietveld analyses were performed, and the results of the XRD Rietveld analyses agreed with the ones from decay curves. In this work, the analyses of the phase fractions are simplified by the monoexponential decay of the pure phases and the biexponential decay of the mixed phase, showing an easy and inexpensive way of studying the phase structures of the materials.

## Figures and Tables

**Table 1:** The refinement parameters of XRD Rietveld analyses.

*E* (kV/cm)	R3c (R Phase)				P4mm (T Phase)				R Factor	
	*a* (Å)	*c* (Å)	*V* (Å^3^)	vol%	*a* (Å)	*c* (Å)	*V* (Å^3^)	vol%	*R_wp_*%	*R_p_*%
0	5.5182	13.5740	357.966	18.88	3.8999	3.9034	59.372	81.12	9.87	7.73
10	5.5184	13.5734	357.980	23.37	3.9005	3.9037	59.392	76.63	9.72	7.70
20	5.5190	13.5755	358.104	46.01	3.9008	3.9040	59.405	53.99	9.07	6.92
30	5.5195	13.5769	358.277	79.00	3.9019	3.9051	59.456	21.00	7.72	5.84
40	5.5199	13.5768	358.260	85.68	3.9020	3.9058	59.470	14.32	7.74	5.47
50	5.5199	13.5769	358.260	87.00	3.9019	3.9051	59.450	13.00	10.86	8.16

Adapted from our earlier work [27].

## Supplementary Materials

The following are available online at https://www.mdpi.com/1996-1944/13/6/1347/s1, Figure S1: The refractive indexes of NKBET10 and NKBET30 ceramics. The refractive indexes were measured by a spectroscopic ellipsometer (V-VASE, J.A. Woollam Co, Lincoln, NE, USA), Table S1: The crystal structure parameters for rhombohedral phase (R3c space group), Table S2: The crystal structure parameters for tetragonal phase (P4mm space group).
