# Supplementary material for: Analyses of Electric Field-Induced Phase Transformation by Luminescence Study in Eu3+-doped (Na, K)0.5Bi0.5TiO3 Ceramics
Source: Materials (Basel). 2020 Mar 16;13(6):1347. doi: 10.3390/ma13061347 (PMC7143870; doi:10.3390/ma13061347)
Supplement: Supplementary file 1 [file materials-13-01347-s001.pdf]

materials

Supplementary Materials

Analyses of Electric Field-Induced Phase Transformation by Luminescence Study in Eu^{3+}-doped $(Na, K)_{0.5}Bi_{0.5}TiO_3$ Ceramics

Liang Zeng and Ji Zhou *

State Key Laboratory of New Ceramics and Fine Processing, School of Materials Science and Engineering, Tsinghua University, Beijing 100084, China; zeng-l15@mails.tsinghua.edu.cn
* Correspondence: zhouji@tsinghua.edu.cn; Tel.: +86-010-6279-5384
Received: 21 February 2020; Accepted: 13 March 2020; Published: date

The non-radiative probability

The probabilities for radiative transitions and non-radiation processes determine the lifetime of an emissive level J, which can be given by:

$$\frac{1}{\tau} = \sum_{J'} A(\Psi J, \Psi J') + W \quad \text{(S1)}$$

where τ is the lifetime of level J; $A(\Psi J, \Psi J')$ is the radiative probability of the transition $\Psi J \rightarrow \Psi J'$; the summation is for transitions which terminate on final level J'; W is the non-radiative probability.

The radiative lifetime τ_{rad} of an emissive level J is the lifetimes in the absence of non-radiation processes:

$$\frac{1}{\tau_{\mathrm{rad}}} = \sum_{J'} A(\Psi J, \Psi J') \quad \text{(S2)}$$

According to Reference 11, the radiative lifetime can be calculated by the following formula:

$$\frac{1}{\tau_{\mathrm{rad}}} = A_{\mathrm{MD}} n^3 \left(I_{\mathrm{total}}/I_{\mathrm{MD}}\right) \quad \text{(S3)}$$

Here A_{MD} is the radiative probability of the transition ${}^5\mathrm{D}_0 \rightarrow {}^7\mathrm{F}_1$ in vacuum, which can be taken as a constant of 14.65 s^{-1}; n is the refractive index of the host; $I_{\mathrm{total}}/I_{\mathrm{MD}}$ is the ratio of the total integral intensity of the ${}^5\mathrm{D}_0 \rightarrow {}^7\mathrm{F}_J$ bands to the integral intensity of the ${}^5\mathrm{D}_0 \rightarrow {}^7\mathrm{F}_1$ band, which can be obtained from the spectrum.

From Equations S1 and S3, the non-radiative probabilities can be calculated. The refractive indexes of NKBET10 and NKBET30 ceramics are shown in Figure S1. The differences of the refractive indexes between NKBET10 and NKBET30 ceramics are so small that can be neglected. In the range of 570–700 nm, the refractive indexes decrease slightly. Here we used the values of 2.52 for convenience. $I_{\mathrm{total}}/I_{\mathrm{MD}}$ for NKBET10 and NKBET30 ceramics can be fitted from Figure 2A, which are 4.741 and 4.109 respectively. Since τ^{R} and τ^{T} are 685.3 μs and 754.1 μs respectively, we obtained $\frac{1}{\tau^{\mathrm{R}}_{\mathrm{rad}}} = 1111.49\mathrm{s}^{-1}$, $\frac{1}{\tau^{\mathrm{T}}_{\mathrm{rad}}} = 963.33\mathrm{s}^{-1}$, $W^{\mathrm{R}} = 347.72\mathrm{s}^{-1}$, $W^{\mathrm{T}} = 362.75\mathrm{s}^{-1}$. Superscripts R and T represent the Eu^{3+} ions in R and T phases respectively, using NKBET10 and NKBET30 ceramics as references respectively.

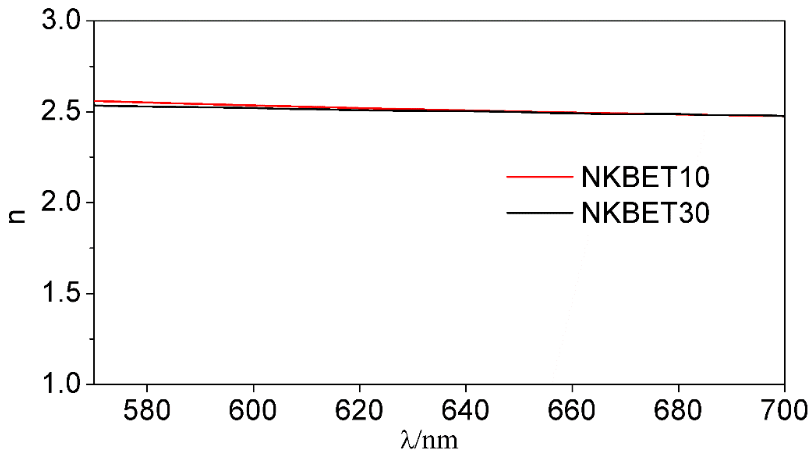

Figure S1. The refractive indexes of NKBET10 and NKBET30 ceramics. The refractive indexes were measured by a spectroscopic ellipsometer (V-VASE, J.A. Woollam Co, Lincoln, NE, USA).

From the values of the radiative probabilities and the non-radiative probabilities, it can be concluded that the radiative transitions dominates the decay processes for the Eu^{3+} ions in both R phase and T phase. Besides, the difference of the non-radiative probabilities between R and T phases is small, and the difference of the radiative probabilities mainly contributes to the variation of the decay processes.

Table S1. The crystal structure parameters for rhombohedral phase (R3c space group).

Atoms	Coordinates			Multiplier	Occupancy
	x	*y*	*z*		
Na	0	0	0.2594	6	0.4
K	0	0	0.2594	6	0.1
Bi	0	0	0.2594	6	0.497
Eu	0	0	0.2594	6	0.003
Ti	0	0	0.0024	6	1
O	0.1295	0.326	0.0833	18	1

Table S2. The crystal structure parameters for tetragonal phase (P4mm space group).

Atoms	Coordinates			Multiplier	Occupancy
	x	*y*	*z*		
Na	0	0	−0.006	1	0.4
K	0	0	−0.006	1	0.1
Bi	0	0	−0.006	1	0.497
Eu	0	0	−0.006	1	0.003
Ti	0.5	0.5	0.5	1	1
O	0.5	0.5	0.016	1	1
O	0.5	0	0.512	2	1

© 2020 by the authors. Submitted for possible open access publication under the terms and conditions of the Creative Commons Attribution (CC BY) license (http://creativecommons.org/licenses/by/4.0/).
